# Correspondence on “Clinical, neuropathological, and genetic characterization of *STUB1* variants in cerebellar ataxias: a frequent cause of predominant cognitive impairment” by Roux et al.

**DOI:** 10.1038/s41436-021-01104-1

**Published:** 2021-02-09

**Authors:** Joohyun Park, Natalie Deininger, Maren Rautenberg, Carsten Saft, Florian Harmuth, Marc Sturm, Olaf Riess, Ludger Schöls, Matthis Synofzik, Tobias B. Haack

**Affiliations:** 1 Institute of Medical Genetics and Applied Genomics, University of Tübingen, Tübingen, Germany; 2 Department of Neurology, Huntington Centre NRW, Ruhr-University Bochum, St. Josef-Hospital, Bochum, Germany; 3 Centre for Rare Diseases, University of Tübingen, Tübingen, Germany; 4 Department of Neurodegenerative Diseases, Hertie Institute for Clinical Brain Research, University of Tübingen, Tübingen, Germany; 5 German Center for Neurodegenerative Diseases (DZNE), Tübingen, Germany

With great interest, we read the publication by Roux et al. about the occurrence of *STUB1* variants in mostly dominant cerebellar ataxias.^1^ We here provide data on heterozygous *STUB1* variants observed in 46 patients in a cohort of 847 ataxia patients, and illustrate the complexity and challenges in the clinical interpretation of heterozygous *STUB1* variants with two case reports.

We screened diagnostic exomes (*n* = 7,832) for rare heterozygous *STUB1* variants (minor allele frequency <0.1% in gnomAD v2.1.1, gnomad.broadinstitute.org) and detected 30 different alterations in 46 ataxia patients (20 females, 26 males). Consistent with the findings by Roux et al., all of them had adult-onset ataxia accompanied by cognitive impairment in 14 cases (median age of onset: 35 years, range 14–66 years).^1^ Predicted protein-truncating variants (PTVs) were identified in 16 (9 females, 7 males) of 847 ataxia patients, while only 3 PTVs were found in 6,985 nonataxia individuals. Additional nonsynonymous variants were identified in 30 ataxia patients (5 in-frame deletions, 25 missense variants) and in 62 individuals (all missense) with unrelated phenotypes. PTVs in *STUB1* were significantly enriched in ataxia cases compared to controls (*p* = 2.17×10^-13^, 16/847 cases vs. 3/6,985 controls, Fisher’s exact test). Enrichment of nonsynonymous *STUB1* variants was less pronounced but exome-wide significant defined as a *p* value of < 1.25×10^-6^, correcting for ~20,000 CCDS genes (*p* = 1.3×10^-8^, 30/847 cases vs. 62/6,985 controls). This finding is in line with the variable functional relevance of, e.g., missense changes or in-frame deletions requiring supportive evidence from either their identification in additional ataxia patients as well as functional validation and/or segregation studies to establish a firm diagnosis.

In patient 1, a 36-year-old male with cerebellar ataxia, mild distal myoclonus, mild spasticity, and cerebellar atrophy, we detected a start-lost variant in *STUB1* (NM_005861:c.3G>A;p.Met1?) by exome sequencing. First clinical features manifested at the age of 30 years. Carrier testing of the parents indicated that the change occurred de novo in the patient. No additional rare variants in ataxia-associated genes were observed.

In patient 2, a 60-year-old female who developed cerebellar ataxia at age of 40 years, we identified a heterozygous frameshift variant (c.689_692del;p.Tyr230CysfsTer9) by exome sequencing. Sanger segregation analysis revealed this variant in her affected father and affected brother, but surprisingly her healthy 56-year-old sister also carried this variant in a heterozygous state. All four members of the family also harbored an additional variant of unknown significance (VUS) in *AFG3L2* (NM_006796:c.2167G>A;p.Val723Met). Variants in *AFG3L2* have been associated with spinocerebellar ataxia type 28 (SCA28).^2^ Notably, Roux et al. identified a patient (family AAD-541) carrying a VUS in *AFG3L2* together with the recurrent missense change p.Tyr49Cys in *STUB1* and suggested that these concomitant changes may have synergic effects. However, the clinical presentation of patient 2 and family AAD-541 match more closely the phenotype of SCA48 patients and characteristic features associated with SCA28 such as ophthalmoplegia have not been observed. To our understanding, the clinical relevance of these *AFG3L2* variants currently remains unclear.

Different PTVs and other nonsynonymous variants have been reported to cause autosomal recessive spinocerebellar ataxia type 16 (SCAR16), autosomal dominant spinocerebellar ataxia type 48 (SCA48), and also both with a possible variable penetrance posing some difficulties for the clinical interpretation and classification of *STUB1* variants.^3–10^ Our findings lend further support to the hypothesis that heterozygous PTVs cause *STUB1-*associated dominant ataxia. However, follow-up segregation analysis of a given variant in available family members remains important to either corroborate a disease-causal role (e.g., a de novo status of the start-lost variant in patient 1) or investigate the possibility of additional factors that might have an impact on penetrance and/or expressivity of heterozygous PTVs in SCA48.^1,4,6^

For missense changes, the interpretation of a putative disease association substantially relies on available allele frequencies in both, disease as well as control cohorts. Examples for variants with statistical support of a disease association include the substitutions p.Tyr49Cys and p.Asn65Ser, which have been found in seven and two ataxia patients, respectively, in our ataxia cohort. For other changes such as the variant p.Arg222Lys reported by Roux et al., we consider the postulated association with the patient’s disease phenotype rather unlikely as this allele is also listed in five individuals in gnomAD (non-neuro cohort) and four individuals without ataxia in our in-house database. However, reduced penetrance of *STUB1* variants has been suggested as an additional mechanism in previous reports. For example four healthy obligate heterozygotes—with two of them deceased after the age of 75 years–were found in three SCA48 families (two with PTVs and one with a missense variant).^1,6^ In addition, the sister of patient 2 reported in this study showed no signs of ataxia by the age of 56 years and there is another heterozygote of this frameshift variant in gnomAD (non-neuro cohort) aged between 50 and 55 years. Furthermore, PTVs have been linked to recessive inheritance patterns as well with heterozygous parents being reportedly asymptomatic.^5,7,10^ Age at onset of SCA48 ranged from 24 to 74 years, demonstrating that some of these heterozygotes may yet develop clinical signs at a later age.^1^ Long-term follow-up of additional healthy heterozygotes is needed to further investigate the age dependency of penetrance. A possible role of gender in disease manifestation and penetrance of *STUB1* variants has been suggested by Roux et al. due to a notable gender imbalance with 70% female cases in their cohort.^1^ However, we could not replicate this finding in our cohort comprising 43% affected females.

In summary, our data provide supportive evidence that heterozygous *STUB1* variants, especially PTVs, cause SCA48. We emphasize a critical evaluation of the pathogenicity of missense variants observed only in single ataxia cases, especially if they are present in controls and lack supportive results from extended segregation analyses. Several lines of evidence point toward the possibility of incomplete penetrance and involvement of additional genetic factors. Comprehensive genetic testing data sets from larger *STUB1* cohorts combined with functional readouts will be crucial to address these questions and provide a robust basis for clinical interpretation of *STUB1* variants and genetic counseling of patients and their families.
